# Supplementary material for: Very Low-Carbohydrate Ketogenic Diet for the Treatment of Severe Obesity and Associated Non-Alcoholic Fatty Liver Disease: The Role of Sex Differences
Source: Nutrients. 2020 Sep 9;12(9):2748. doi: 10.3390/nu12092748 (PMC7551320; doi:10.3390/nu12092748)
Supplement: Supplementary file 1 [file nutrients-12-02748-s001.zip › Supplementary/Supplementary_Table_1.docx]

**Supplementary Table 1.** Association of sex and menopausal state with improvement of Edmonton stage and ultrasonographic degree of steatosis (Fisher’s exact test). OR is calculated for improvement vs no improvement.

|  | **Category** | **Reference** | ***OR*** | **95% *CI*** | ***p*** |
| --- | --- | --- | --- | --- | --- |
| **Edmonton stage** | Females | Males | 5.714 | 0.652-50.00 | 0.132 |
|  | Pre-menopausal females | Males | 9.345 | 0.957-90.90 | 0.080 |
|  | Post-menopausal females | Males | 3.225 | 0.297-35.71 | 0.600 |
|  | Post-menopausal females | Pre-menopausal females | 0.346 | 0.068-1.759 | 0.252 |
| **Steatosis degree** | Females | Males | 0.353 | 0.099-1.254 | 0.132 |
|  | Pre-menopausal females | Males | 0.303 | 0.069-1.328 | 0.156 |
|  | Post-menopausal females | Males | 0.404 | 0.097-1.675 | 0.296 |
|  | Post-menopausal females | Pre-menopausal females | 1.333 | 0.327-5.434 | 0.735 |
