# Supplementary figures and images for: Very Low-Carbohydrate Ketogenic Diet for the Treatment of Severe Obesity and Associated Non-Alcoholic Fatty Liver Disease: The Role of Sex Differences
Source: Nutrients. 2020 Sep 9;12(9):2748. doi: 10.3390/nu12092748 (PMC7551320; doi:10.3390/nu12092748)

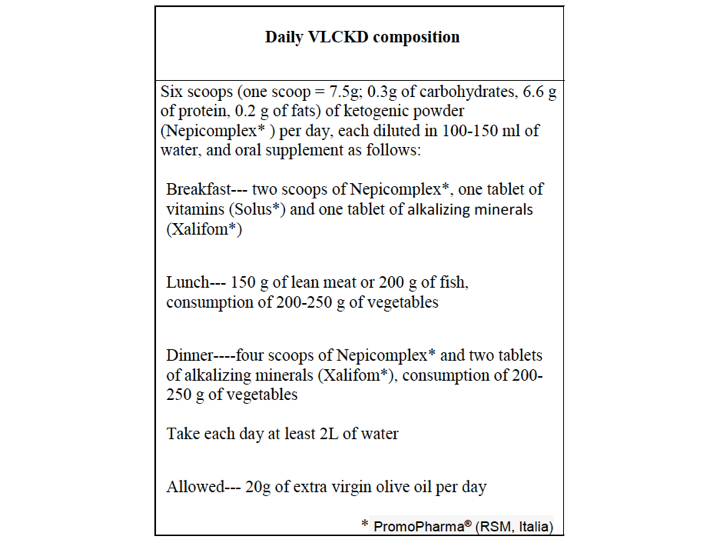

Supplement: Supplementary file 1 [file nutrients-12-02748-s001.zip › Supplementary/Supplementary_Figure_1.tiff]
